# Untargeted Metabolomic Profiling of Cuprizone-Induced Demyelination in Mouse Corpus Callosum by UPLC-Orbitrap/MS Reveals Potential Metabolic Biomarkers of CNS Demyelination Disorders

**DOI:** 10.1155/2021/7093844

**Published:** 2021-09-14

**Authors:** Zhijie Zhao, Tongqi Li, Xiaohua Dong, Xiaojing Wang, Zhongxiao Zhang, Changyi Zhao, Xueran Kang, Ruizhe Zheng, Xinyuan Li

**Affiliations:** ^1^Department of Neurosurgery, Tongren Hospital, Shanghai Jiao Tong University School of Medicine, Shanghai 200336, China; ^2^Department of Ophthalmology, Shanghai General Hospital, Shanghai Jiao Tong University School of Medicine, Shanghai 200940, China; ^3^Hongqiao International Institute of Medicine, Tongren Hospital, Shanghai Jiao Tong University School of Medicine, Shanghai 200336, China; ^4^Department of Pediatrics, Jingjiang People's Hospital Affiliated to Yangzhou University, Jingjiang 214500, China; ^5^Department of Otorhinolaryngology Head and Neck Surgery, Shanghai Ninth People's Hospital, Shanghai Jiao Tong University School of Medicine, Shanghai 200011, China

## Abstract

Multiple sclerosis (MS) is a neurodegenerative disorder characterized by periodic neuronal demyelination, which leads to a range of symptoms and eventually to disability. The goal of this research was to use UPLC-Orbitrap/MS to identify validated biomarkers and explore the metabolic mechanisms of MS in mice. Thirty-two C57BL/6 male mice were randomized into two groups that were fed either normal food or 0.2% CPZ for 11 weeks. The mouse demyelination model was assessed by LFB and the expression of MBP by immunofluorescence and immunohistochemistry. The metabolites of the corpus callosum were quantified using UPLC-Orbitrap/MS. The mouse pole climbing experiment was used to assess coordination ability. Multivariate statistical analysis was adopted for screening differential metabolites, and the ingenuity pathway analysis (IPA) was used to reveal the metabolite interaction network. We successfully established the demyelination model. The CPZ group slowly lost weight and showed an increased pole climbing time during feeding compared to the CON group. A total of 81 metabolites (VIP > 1 and *P* < 0.05) were determined to be enriched in 24 metabolic pathways; 41 metabolites were markedly increased, while 40 metabolites were markedly decreased in the CPZ group. The IPA results revealed that these 81 biomarker metabolites were associated with neuregulin signaling, PI3K-AKT signaling, mTOR signaling, and ERK/MAPK signaling. KEGG pathway analysis showed that two significantly different metabolic pathways were enriched, namely, the glycerophospholipid and sphingolipid metabolic pathways, comprising a total of nine biomarkers. Receiver operating characteristic analysis showed that the metabolites (e.g., PE (16 : 0/22 : 6(4Z, 7Z, 10Z, 13Z, 16Z, 19Z)), PC (18 : 0/22 : 4(7Z, 10Z, 13Z, 16Z)), cytidine 5′-diphosphocholine, PS (18 : 0/22 : 6(4Z, 7Z, 10Z, 13Z, 16Z, 19Z)), glycerol 3-phosphate, SM (d18 : 0/16 : 1(9Z)), Cer (d18:1/18 : 0), galabiosylceramide (d18:1/18 : 0), and GlcCer (d18:1/18 : 0)) have good discrimination ability for the CPZ group. In conclusion, the differential metabolites have great potential to serve as biomarkers of demyelinating diseases. In addition, we identified metabolic pathways associated with CPZ-induced demyelination pathogenesis, which provided a new perspective for understanding the relationship between metabolites and CNS demyelination pathogenesis.

## 1. Background

Multiple sclerosis (MS) is a multifactorial autoimmunity disease of the central nervous system (CNS) that is characterized by the loss of oligodendrocytes and myelin sheaths in the white matter tracts [1]. Inflammation is the main driver of the condition, and oxidative stress contributes to tissue damage and facilitates the existing inflammatory response [2]. Clinical deficits include numbness, muscle spasms, ataxia, paraparesis or hemiparesis, vision loss, pain, and cognitive deficits [3]. These clinical deficits have a major economic impact on medical and health care systems. To date, as a multifactorial disease, the exact pathological mechanism of MS is not known, and the diagnosis of MS remains a challenge [4]. Therefore, it is important to identify efficient biomarkers of MS using new methods to make diagnosis easier and more reliable.

Metabolomics is an emerging discipline that quantifies the low-molecular-weight metabolites of an organism or cell [5]. It is extensively used to diagnose a variety of diseases, such as CNS disorders. Metabolites are defined as low-molecular-weight (<1000 Da) molecules [6]. Since the diversity of the metabolite composition leads to a series of physicochemical properties, metabolomic profiling has the potential to provide information that cannot be gleaned from other technologies, such as genomics, transcriptomics, or proteomics. Liquid chromatography-mass spectrometry (UPLC-Orbitrap/MS) analysis is the most popular technique for identifying and quantifying metabolites and allows a minute profiling of metabolites identified in biological systems, revealing the characterization of dynamic multiparameter responses to diverse endogenous and exogenous processes while assessing epigenetic modifications [7]. Many scholars have studied metabolite changes in demyelinated brain tissue in cerebrospinal fluid and blood [4, 8], but direct studies of demyelinated brain tissue are relatively lacking.

Cuprizone (CPZ) is a copper chelator that targets many metalloenzymes, and sustained ingestion results in white matter lesions similar to those of model III MS lesions [9], accompanied by widespread macrophage/microglia activation, oligodendrocyte apoptosis, and demyelination [10]. Therefore, it is widely used in experimental approaches based on animal models to study the mechanisms of demyelination [11] and cellular responses [12]. The pathological response of C57BL/6 mice after CPZ poisoning has high reproducibility and good characteristics. In this model, demyelination was evident after intoxication in a variety of structures including the hippocampus, external capsule [13], cerebellar cephalic peduncle [14], cerebellum [15], striatum, cerebral cortex [16], and, most notably, the corpus callosum [17, 18]. The corpus callosum has previously been shown to be the area most affected by CPZ.

Although several studies have been performed using the CPZ model to explore the molecular mechanisms of corpus callosum demyelination [19, 20], the metabolic pathways and differential metabolites remain to be elucidated. The present study used UPLC-Orbitrap/MS to analyze corpus callosum specimens of the CPZ model to identify the key metabolic biomarkers and mechanisms of CNS demyelinating disorders.

## 2. Methods

### 2.1. Animals and Experimental Design

Male C57BL/6 mice (8 weeks old) were purchased from Spelford (Beijing) Biotechnology Corporation. Mice were housed at 22°C ± 1°C in an air-conditioned space with a 12-hour light and dark cycle (lights on from 7:00 am to 7:00 pm). Food and tap water were available ad libitum. All procedures for subsequent experiments were approved by the Animal Care and Use Committee of the Tongren Hospital, Shanghai Jiao Tong University School of Medicine and were conducted in accordance with the NIH Guide for the Care and Use of Laboratory Animals. All efforts were made to minimize animal distress and discomfort and to reduce the number of animals used.

Thirty-two C57BL/6 male mice (eight weeks old) were randomly divided into the normal (*n* = 16) and model groups (*n* = 16), and the mice in the model group were fed a diet containing 0.2% cuprione (reagents purchased from Sigma, custom-made diet from Jiangsu Synbiotic Feeds). By referring to the literature [21], C57BL/6 male mice were selected to be fed 0.2% cuprizone chow to construct the demyelination model, and we chose 11 weeks of continuous feeding for the CPZ group. Mouse body weight was measured every week. The pole climbing experiment was performed once a week. Next, the mice were sacrificed, and the corpus callosum was immediately collected for demyelination identification and UPLC-Orbitrap/MS analysis. Of these, six pairs of mice were selected for demyelination validation (3 pairs for western blotting and 3 pairs for Luxol fast blue staining, immunofluorescence, and immunohistochemistry), and 10 pairs were used for UPLC-Orbitrap/MS.

### 2.2. Western Blotting

The corpus callosum was lysed for total protein extraction by RIPA lysate I (C500005, Sangon Biotech, China). The protein concentration was determined by the BCA Protein Assay Kit (C503021, Sangon Biotech, China). Sodium dodecyl sulfate-polyacrylamide gel electrophoresis (SDS-PAGE, P0015L, Beyotime, China) was used to separate the proteins (25 *μ*g) from each sample, which were then electrophoretically transferred to polyvinylidene fluoride membranes by electrophoresis (PVDF, IPVH00010, Millipore, USA). The membrane was blocked in 5% skim milk in Tris-buffered saline solution, pH 7.4, for 1 hour at room temperature and incubated with the primary antibody at 4°C overnight. The following antibodies were used: myelin, anti-myelin basic protein (MBP, 1 : 100, Abcam, Cambridge, MA, USA), anti-glial fibrillary acidic protein (GFAP, 1 : 100, Abcam, Cambridge, MA, USA), Alexa Fluor 555 goat anti-rabbit IgG (H+L) (1 : 50, A0208, Beyotime, China), and Alexa Fluor 488 goat anti-mouse IgG (H+L) (1 : 50, A0216, Beyotime, China). On the second day, the membranes were moved out and restored to room temperature, followed by rinsing of the membranes in PBST buffer 3 times/5 min. Next, 3 mL of secondary antibody solution was added, and the membrane was incubated for 2 h at room temperature and rinsed in PBST buffer 3 times/5 min. The proteins were determined using an ECL western blot detection kit (36208ES76, Yeasen, China). Then, the exposure results were read by placing them in the instrument.

### 2.3. Immunohistochemistry

Paraffin-embedded samples were dewaxed and subjected to antigen retrieval in 10 mM citrate buffer (pH 6.0). The primary antibodies used for immunostaining were as follows: anti-glial fibrillary acidic protein (GFAP, 1 : 100, Abcam, Cambridge, MA, USA) and anti-myelin basic protein (MBP, 1 : 100, Abcam, Cambridge, MA, USA). All antibodies were diluted in PBS containing 0.5% Triton X-100. Sections were further incubated with biotinylated secondary antibodies.

### 2.4. Double Immunofluorescence

The sections were incubated with Alexa Fluor 555 goat anti-rabbit IgG (H+L) (1 : 50, A0208, Beyotime, China) and Alexa Fluor 488 goat anti-mouse IgG (H+L) (1 : 50, A0216, Beyotime, China). Brain sections were analyzed with Mayer's hemalum solution (Merck, Darmstadt, Germany) for DAB cell analysis or immunofluorescence staining with 4′,6-diamidino-2-phenylindole dihydrochloride (DAPI, E607303, Sangon Biotech, China).

### 2.5. LFB Myelin Staining

Luxol fast blue (LFB) is a copper phthalide beet dye with staining properties that binds to myelin phospholipids in alcoholic solutions. The application of LFB myelin staining can show the structure of myelin in nervous tissue very well. The test was performed in samples that were randomly selected from the normal and model groups of mice. The brain was harvested, and the corpus callosum region was sectioned for LFB staining.

### 2.6. Sample Extraction

Approximately 50 mg of tissue was added to 0.5 mL of solvent (methanol : water = 8 : 2) containing an internal standard (4 *μ*g/mL, 2-chloro-L-phenylalanine), homogenized, and crushed. The homogenized tissues were placed in a centrifuge (13,000 rpm, 4°C) for 10 min, and 200 *μ*L of supernatant was placed into the injection vial for subsequent metabolomic analysis. In addition, an equal volume (approximately 20 *μ*L) of each sample to be tested was taken and mixed as a QC (quality control) sample.

### 2.7. Lipid Analysis by UPLC-Orbitrap/MS

UPLC-Orbitrap/MS analysis was performed using an Ultimate 3000 ultraperformance liquid chromatograph coupled with a Thermo Scientific Orbitrap Elite mass spectrometer. The chromatographic column was a Kinetex C18 (100 × 2.1 mm, 1.9 *μ*m). The mobile phase was (a) 0.1% formic acid solution and (b) acetonitrile (0.1% formic acid); the flow rate was 0.4 mL/min; the column temperature was 25°C; the post time was 5 min; and the injection volume was 3 *μ*L. The optimized chromatographic gradient was as follows: 0–2 min, 5% B; 2–13 min; and the post time was set to 5 min to equilibrate the system.

After ultra-HPLC isolation, the specimens were analyzed using a Q Thermo Orbitrap Elite mass spectrometer. The parameters of the positive ion mode were as follows: heater temperature 300°C, sheath gas flow rate 45 arb, aux gas flow rate 15 arb, sweep gas flow rate 1 arb, spray voltage 3.0 kV, capillary temperature 350°C, and S-lens RF level 30%. The scan range was 200–1500. For the negative ion mode, the heater temperature was 300°C, sheath gas flow rate 45 arb, aux gas flow rate 15 arb, sweep gas flow rate 1 arb, spray voltage 2.5 kV, capillary temperature 350°C, and S-lens RF level 60%. The scan range was 200–1500.

### 2.8. Statistical Analyses

Receiver operating characteristic (ROC) curve analysis was used to calculate the sensitivity and specificity of the variables. The performance of the biomarkers was quantified by the area under the receiver operating characteristic curve (AUC), and an AUC greater than 0.75 indicates that the marker has good discrimination.

Thermo's own software compound discovery was used to complete the extraction of compound components in the sample and for data preprocessing, including baseline filtering, peak identification and integration, retention time correction, and peak alignment and mass spectral fragment attribution analysis, and finally, postediting was achieved using Excel 2016 software, including impurity peaks from column loss and sample preparation caused by removal and quantitative ion selection. The final results were organized into a two-dimensional data matrix including the variables and peak intensity values (normalization process) to finally obtain the compound structure information. The edited data matrix was imported into Simca-P software (version 11.0) and processed for multivariate statistical analysis after centralization and Pareto scalarization. Differential metabolites were distinguished based on the combination of statistical thresholds of variable influence on projection (VIP) obtained from the PLS-DA model (multidimensional statistical analysis) and a two-sided Student's *t* test (*P* value < 0.05) on the raw data (unidimensional statistical analysis). MedCalc statistical software (version 19.05) was used to perform receiver operating characteristic (ROC) analysis and to calculate the area under the ROC curve (AUC). In addition, other statistical analyses were performed with R software (version 3.5.0), GraphPad Prism (version 8), TBtools software (version 1.0), and SPSS software (version 25, IBM Corp.). *P* values less than 0.05 were considered statistically significant.

## 3. Results

### 3.1. Evaluation of the Cuprizone-Induced Demyelinated-Like Mouse Model

During the experiment, LFB was used to assess the degree of demyelination in the corpus callosum of the brain. Immunohistochemistry, immunofluorescence, and western blotting were used to detect MBP and glial fibrillary acidic protein (GFAP) expression and finally combined with behavioral testing to explore the successful establishment of the demyelination model.

### 3.2. Complete Demyelination of the Corpus Callosum

**Figure 1:**
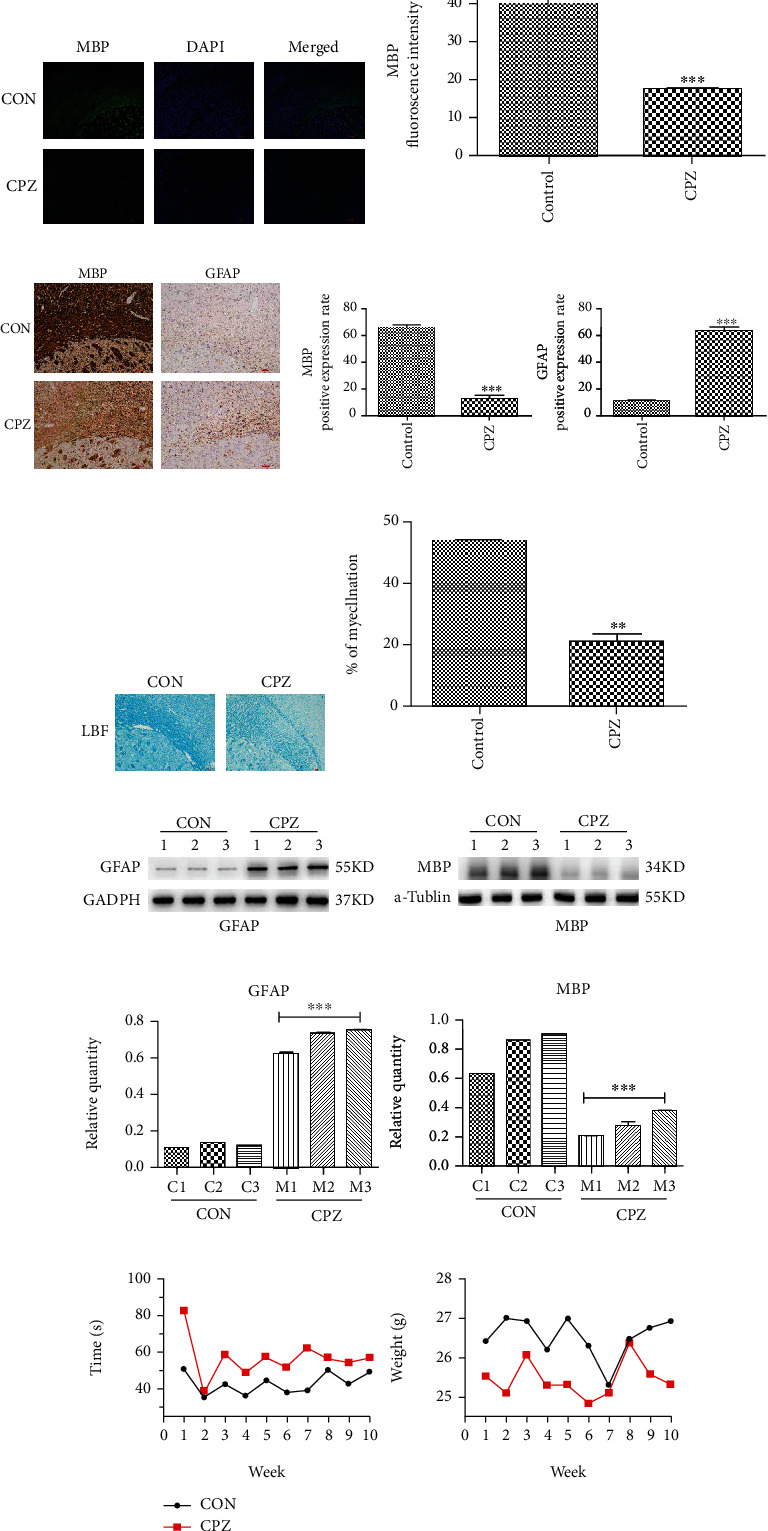
The immunofluorescence evaluation of MBP as a marker of myelin. (a) Representative images (×20 field) of the corpus callosum sections showing myelin (MBP, green) and nuclei (DAPI, blue). (b) Quantification of immunofluorescence data showed that changes in the expression of MBP were significant between studied groups. (c) Immunohistochemistry evaluation of MBP and GFAP, representative images (×40 field) of the corpus callosum. (d) Quantification of immunohistochemistry data showed that changes in the expression of MBP and GFAP were significant between studied groups. (e) Demyelination of the corpus callosum in CPZ mice as shown by myelin staining with LFB (×40 field). (f) Quantitative analysis of LFB images showed changes of myelin in the corpus callosum between the CPZ mouse and the control mouse. (g) Western blotting showing the protein levels of MBP and GFAP in the corpus callosum between the CPZ mouse and the control mouse. (h) Bar graphs showing the quantification of protein levels of MBP and GFAP between the CPZ mouse and the CON mouse. (i) Weight statistics: all mice gain weight as time increases and the CPZ group had lower weight than the CON group; pole climbing time statistics: the CPZ group had longer pole climbing time than the CON group. The values are expressed as mean ± SEM. Significance is indicated by ^∗∗∗^*P* ≤ .001, ^∗∗^*P* ≤ .01, and ^∗^*P* ≤ .05. DAPI: 4′,6-diamidino-2-phenylindole dihydrochloride; MBP: myelin basic protein; GFAP: glial fibrillary acidic protein; CON: control; CPZ: cuprizone; LFB: Luxol fast blue.

Immunofluorescence indicated that MBP expression was downregulated in the CPZ group (Figures 1(a) and 1(b)). Immunohistochemistry revealed that the expression of MBP in the CPZ group was significantly reduced compared to that in the CON group, and the expression of GFAP in the CPZ group was increased (Figures 1(c) and 1(d)). In addition, the corpus callosum region was sectioned for LFB staining. In the CPZ demyelination model, the corpus callosum region was lightly stained (Figures 1(e) and 1(f)). The protein expression of MBP and GFAP was detected by western blotting in the CPZ group and the CON group (Figures 1(g) and 1(h)). The results showed that the CPZ group slowly lost weight and had an increased pole climbing time during feeding compared to the CON group (Figure 1(i)).

### 3.3. Untargeted UPLC-Orbitrap/MS-Based Metabolomic Analysis of the Corpus Callosum

#### 3.3.1. Overall PCA and System Stability Examination

**Figure 2:**
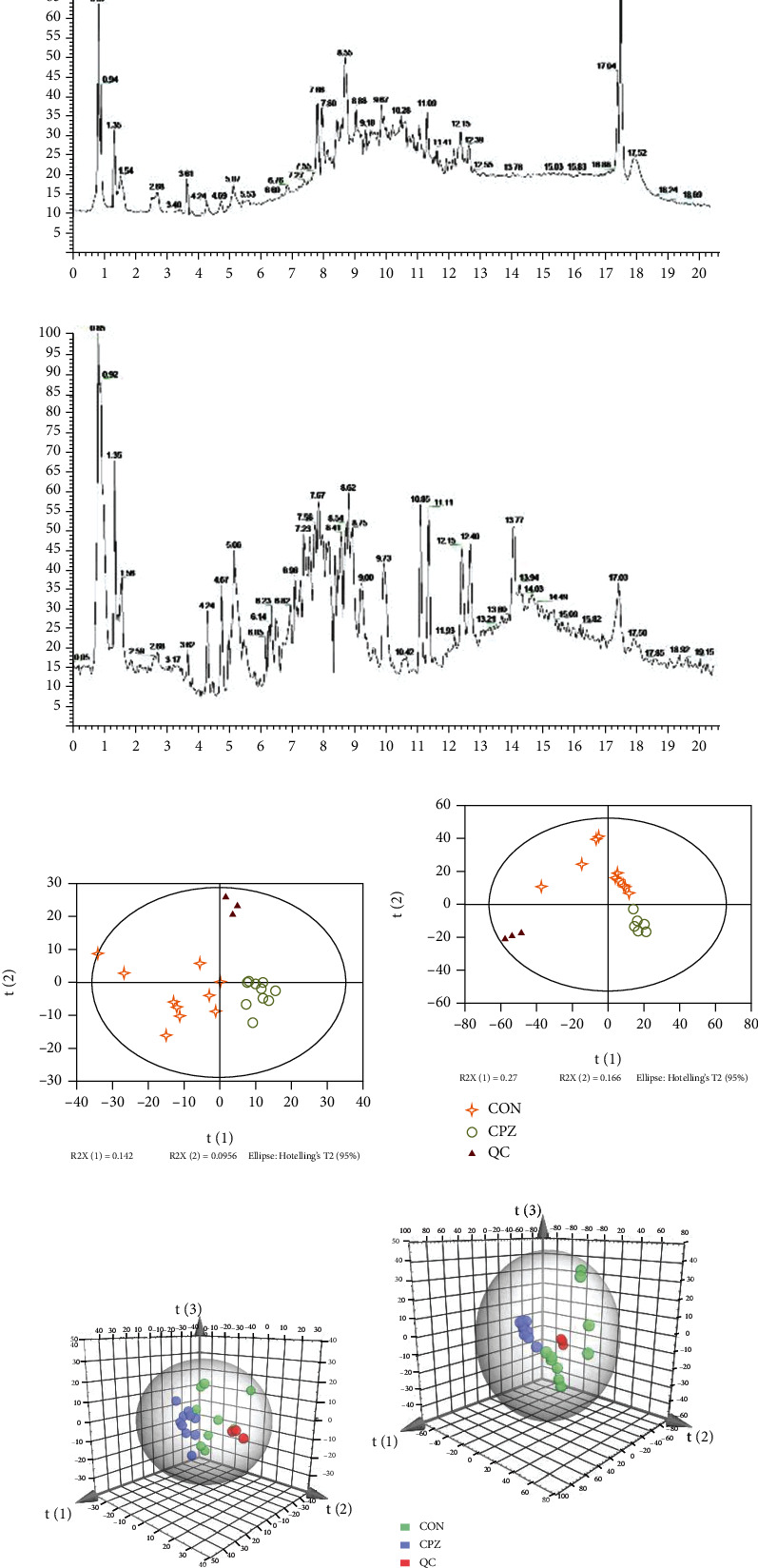
(a) TIC (pos), (b) TIC (neg), (c) PCA score (pos), (d) PCA score (neg), (e) PCA 3D score plot (pos), and (f) PCA 3D score plot (neg). TIC: total ion flow chromatogram; PCA: principal component analysis; pos: positive; neg: negative.

In the subsequent analysis, the data were checked for completeness and no missing values were found. The total ion flow chromatogram (TIC) is shown below Figures 2(a) and 2(b). The principal component analysis (PCA) modeling method was used to examine the degree of aggregation of the samples. PCA is an unsupervised model analysis method that can more reliably reflect the most real differences between groups. This analysis obtained a total of 2 principal components in the positive mode with R[1]X1 = 0.142 and R[2]X2 = 0.0956 and 2 principal components in the negative mode with R[1]X1 = 0.27 and R[2]X2 = 0.166. According to the overall PCA score plot (Figures 2(c)–2(f)), there was a clear trend of separation between the disease and control groups in the positive and negative ion models, indicating significant metabolic differences between the two groups. In addition, all QC injections were tightly clustered in the PCA space. The uniformity of the repeated QC injections and the reliable data quality of all samples demonstrate the validity of the method for metabolic characterization in experiments.

#### 3.3.2. PCA between the CON and CPZ Groups

**Figure 3:**
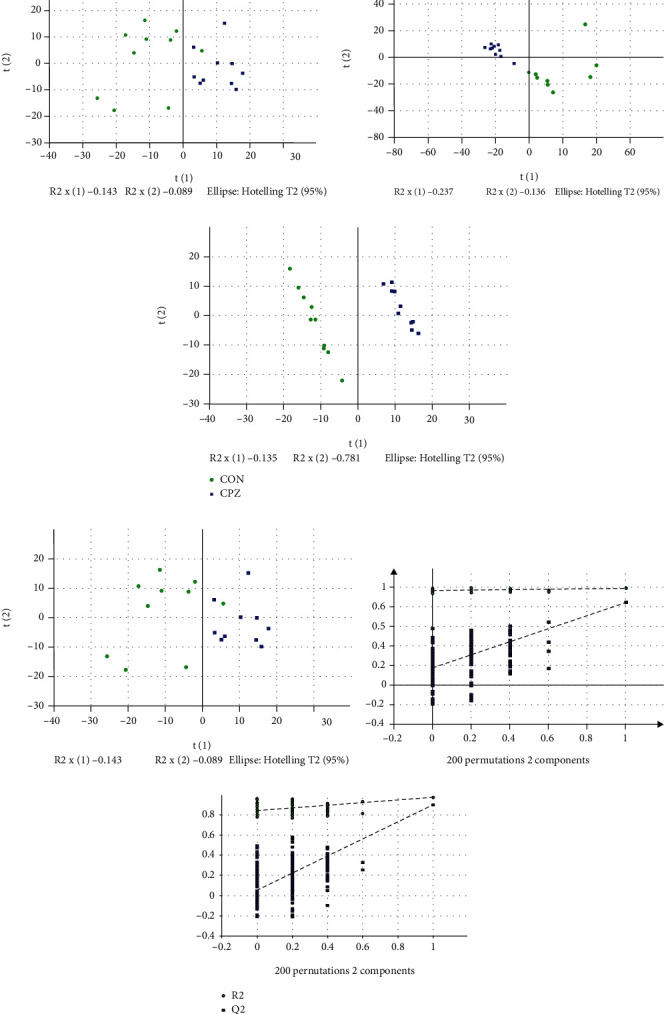
Multivariate statistical analysis of corpus callosum metabolomics for the studied group: (a) PCA Score (pos), (b) PCA Score (neg), (c) PLS-DA score (pos), (d) PLS-DA score (neg), (e) PLS-DA ranking validation (pos), and (f) PLS-DA ranking validation (neg). PLS-DA: partial least squares discriminant analysis; PCA: principal component analysis; pos: positive; neg: negative.

This analysis obtained a total of 2 principal components in the positive mode with cumulative R2X = 0.232 and Q2 = 0.00545 and a total of 2 principal components in negative mode with cumulative R2X = 0.373 and Q2 = 0.0987. The PCA score plot is shown in Figures 3(a) and 3(b), and the oval is the confidence interval, where the horizontal coordinate *t* [1] is the first principal component (PC1) and the vertical coordinate *t* [2] is the second principal component (PC2). According to the overall PCA score plot, there was a clear trend of separation between the CPZ and CON groups in the positive and negative ion modes, indicating significant metabolic differences between the two groups.

#### 3.3.3. PLS-DA between the CON and CPZ Groups

To determine the ion peaks that have the potential to be used to distinguish the metabolite profiles between the two groups, we created a supervised partial least squares discriminant analysis (PLS-DA) model that focused on the actual class-distinguishing variations. The PLS-DA score plot of the 2 groups in the positive and negative modes (Figures 3(c) and 3(d)) showed a clear trend of separation between the groups. A total of 2 principal components were obtained for this analysis in the positive mode, with cumulative R2X = 0.212, R2Y = 0.996, and Q2 = 0.852; a total of 2 principal components were obtained in the negative mode, with cumulative R2X = 0.338, R2Y = 0.972, and Q2 = 0.896. The PLS-DA ranking validation (Figures 3(e) and 3(f)) shows that the model performs well.

#### 3.3.4. Screening of Differential Metabolites and Pathway Analysis

**Figure 4:**
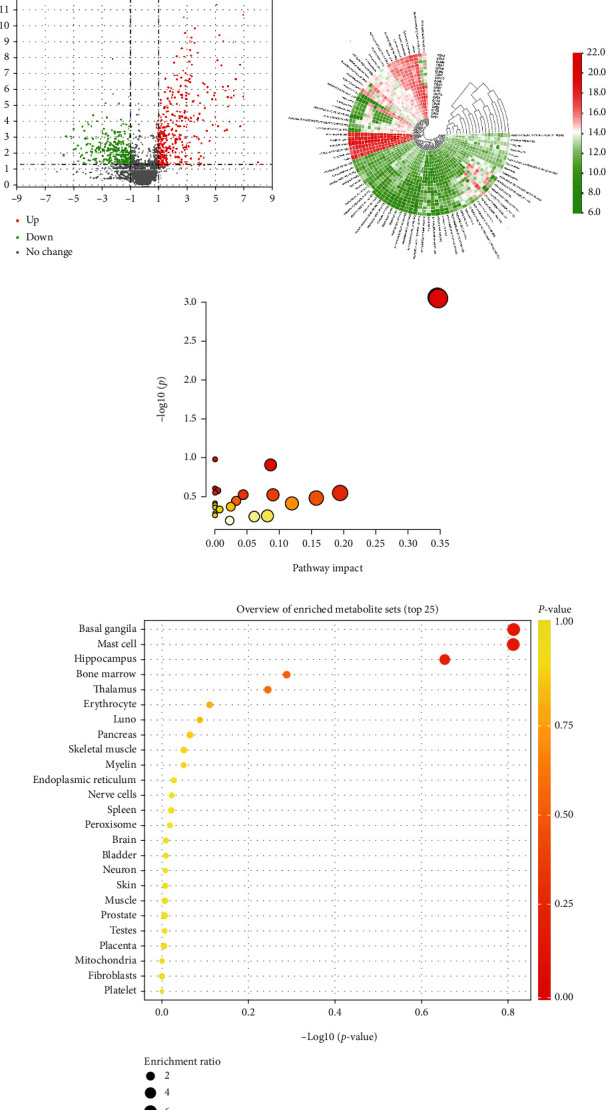
Representative volcano plot (fold change threshold = 1.5 and *P* value in differential metabolites) (a). Representative heat map of 81 differential metabolites between the CPZ group and the CON group (b). KEGG pathway analysis of differential metabolites (c). Enrichment analysis based on HMDB database was used to find the top 10 location items (organ, tissue, and subcellular localizations) with the highest enrichment of differential metabolites (d).

Based on the PLS-DA model, a total of 81 differential metabolites were characterized by KEGG and HMDB databases with VIP > 1 and *P* < 0.05. A total of 41 metabolites were increased, and 40 were decreased (Figure 4(a)). The 81 differential metabolites were used to build a heat map between the CPZ group and the CON group, and the difference in metabolite abundance profiles was illustrated (Figure 4(b)). We performed enrichment analysis by the HMDB database to find the top 10 location items (organ, tissue, and subcellular localizations) with the highest enrichment of differential metabolites, and the results revealed that these 10 subcellular locations were the basal ganglia, mast cells, hippocampus, bone marrow, thalamus, erythrocytes, lung, pancreas, skeletal muscle, and myelin (Figure 4(d)).

**Figure 5:**
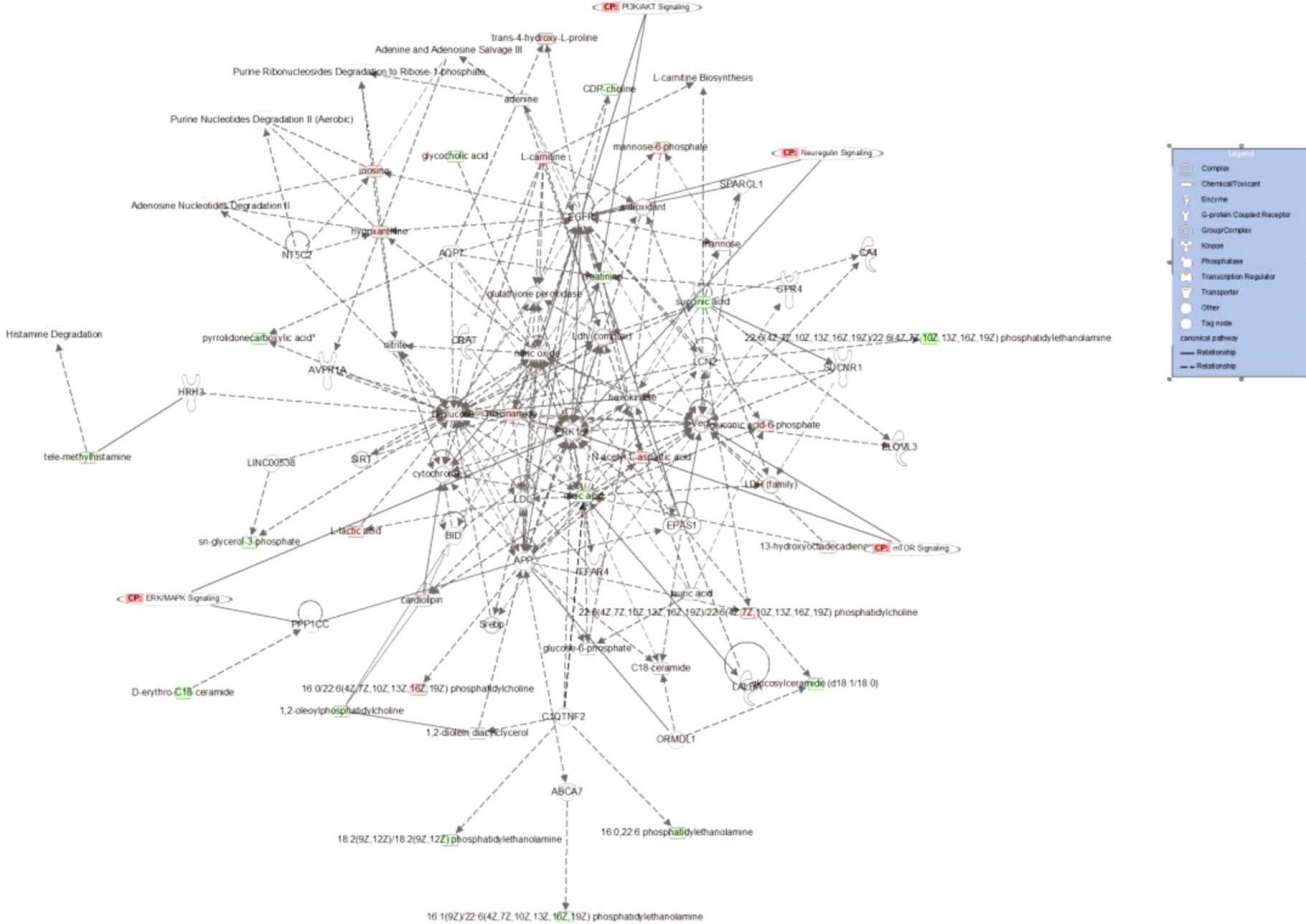
Network and functional analysis of differential metabolites using the IPA database. Red nodes represent upregulated metabolites. Green nodes represent downregulated metabolites. CP represents the signaling pathways associated with the altered metabolites. IPA: ingenuity pathway analysis.

The KEGG pathway analysis showed two significantly different (raw *P* < 0.05) metabolic pathways that were enriched in the CPZ group compared to the CON group; the pathways included glycerophospholipid metabolism and sphingolipid metabolism (Figure 4(c)). In addition, IPA network analysis (Figure 5) showed that these differential metabolites were associated with neuregulin-signaling, PI3K-AKT signaling, mTOR signaling, and ERK/MAPK signaling. The glycerophospholipid metabolism pathway includes five different metabolites: PE (16 : 0/22 : 6(4Z, 7Z, 10Z, 13Z, 16Z, 19Z)), PC (18 : 0/22 : 4(7Z, 10Z, 13Z, 16Z)), cytidine 5′-diphosphocholine, PS (18 : 0/22 : 6(4Z, 7Z, 10Z, 13Z, 16Z, 19Z)), and glycerol 3-phosphate. The sphingolipid metabolism pathway includes four different metabolites: SM (d18 : 0/16 : 1(9Z)), Cer (d18 : 1/18 : 0), galabiosylceramide (d18 : 1/18 : 0), and GlcCer (d18 : 1/18 : 0)).

**Figure 6:**
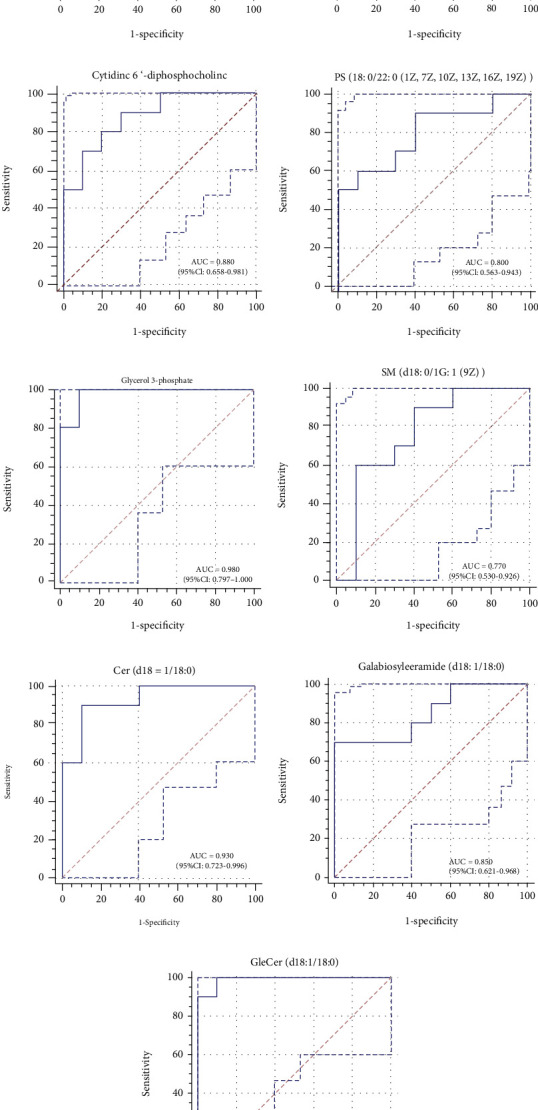
(a–i) The ROC curves show the sensitivity and specificity of the differential metabolites for the prediction of the demyelination. ROC: receiver operating characteristic; AUC: area under curve.

ROC analysis was used to determine the discrimination ability of these different metabolites, and the performances of these predictors were PE (16 : 0/22 : 6(4Z, 7Z, 10Z, 13Z, 16Z, 19Z)) AUC = 0.910, PC (18 : 0/22 : 4(7Z, 10Z, 13Z, 16Z)) AUC = 0.830, cytidine 5′-diphosphocholine AUC =0.880, PS (18 : 0/22 : 6(4Z, 7Z, 10Z, 13Z, 16Z, 19Z)) AUC = 0.800, glycerol 3-phosphate AUC = 0.980, SM (d18 : 0/16 : 1(9Z)) AUC = 0.770, Cer (d18 : 1/18 : 0) AUC = 0.930, galabiosylceramide (d18 : 1/18 : 0) AUC = 0.850, and GlcCer (d18 : 1/18 : 0) AUC = 0.990. The above results showed that these different metabolites have good discrimination for demyelination in our research (Figures 6(a)–6(i)).

## 4. Discussion

Neurodegenerative diseases are caused by the loss of myelin sheaths and/or neurons, which deteriorate and become dysfunctional over time. Destruction of the myelin sheath around the axon leads to various neurological diseases, including MS [22]. A total of approximately 2 million people worldwide are plagued by MS. MS is an inflammatory and demyelinating disease of the CNS. It results in a series of pathological changes caused by the combination of peripheral immune cells and brain resident cells. Among them, oxidative stress and mitochondrial dysfunction are the main causes of demyelination [2]. Previous studies have suggested that the pathology and immune mechanisms of MS are driven by multifaceted mechanisms involving adaptive immunity and oxidative damage; however, the exact etiology of MS remains unclear. The diagnosis and treatment of MS remain a challenge in clinical practice, so identifying validated biomarkers and exploring the molecular mechanisms involved are key factors but are challenging in clinical decision-making. The identification of biomarkers can be used clinically for precise diagnosis and prognosis prediction and to guide treatment options as well as disease monitoring. In recent years, individualized precision therapy has been a hot topic and a area in clinical treatment; thus, methods such as metabolomics, proteomics, and epigenomics have emerged as tools to capture the complex pathological changes in MS [6]. In particular, metabolomics provides detailed analyses of metabolites detected in biological systems, revealing the characterization of dynamic multiparametric responses to various endogenous and exogenous processes while assessing epigenetic changes in diseases [23].

Untargeted metabolomics could be a high-throughput technology that enables simultaneous semiquantitative measurement of different metabolite species in complex samples, such as biological tissues, creating the potential to obtain a comprehensive read of the practical state of the human organism [24]. Among the existing analytical techniques, UPLC-Orbitrap/MS allows the separation of polar, ionized, nonvolatile, and thermally unstable compounds with a broader range of applications. In addition, UPLC-Orbitrap/MS has good selectivity and sensitivity, for both molecules and ions, and more detailed and precise multilevel mass spectrometric information, which enables the identification of unknown metabolites. Metabolomic studies have emerged as a hopeful approach to identify potential biomarkers of MS; however, previous metabolomic studies were based only on indirect studies of demyelinated brain tissue in the cerebrospinal fluid and blood [25, 26]. Although blood metabolite markers for neuronal myelin damage can significantly improve our ability to understand the mechanism of MS and quantify neurodegeneration, the ability to identify demyelinating metabolites is limited. Current studies on the variation in metabolites in demyelinated tissue of the mouse corpus callosum are inadequate. Therefore, we propose the use of UPLC-Orbitrap/MS to identify differential metabolites and related pathways in demyelinated corpus callosum tissue and explore the feasibility of differential metabolites as biomarkers for identifying CPZ models.

The pathological similarities between the CPZ model and MS are reflected in the loss of myelin and axons, activation of the oxidative stress pathway, and the relative integrity of the blood-brain barrier [27], which have been widely used to establish demyelinated mouse models. CPZ is a copper chelator that impairs cytochrome oxidase activity, decreases oxidative phosphorylation, and causes degenerative changes in oligodendrocytes, leading to toxic demyelination resembling that seen in MS patients, with weight loss and behavioral abnormalities [28]. In this study, the results of western blotting, immunofluorescence, immunohistochemistry, and LFB myelin staining showed that C57BL/6 male mice had severe loss of myelin protein in the corpus callosum after 11 weeks of CPZ feeding, which was consistent with previous reports and demonstrated that we successfully replicated the CPZ-induced model of corpus callosum demyelination in C57BL/6 mice. In addition, the body weight of mice within the experimental group was significantly lower, while the length of pole climbing was significantly increased compared to that in the control group. Subsequently, the differential metabolites of the mouse corpus callosum were identified and analyzed for metabolic pathways by UPLC-Orbitrap/MS, and the differential metabolites were annotated by the KEGG and HMDB databases. A total of 81 differential metabolites were successfully annotated. The IPA network showed that these 81 differential metabolites were associated with neuregulin signaling [29], PI3K-AKT signaling [30], mTOR signaling [31], and ERK/MAPK signaling [32], and all four pathways were associated with neurodegenerative diseases. Among them, PI3K-AKT signaling [33], mTOR signaling [34], and ERK/MAPK signaling [35] are associated with oxidative stress. Previous studies have found that CPZ can interfere with the antioxidant system and the electron transport chain, causing a decrease in antioxidant enzymes such as superoxide dismutase (SOD) and glutathione (GSH), and leading to mitochondrial dysfunction and oxidative stress in the corpus callosum [36]. Notably, reduced cellular energy resulting from CPZ-induced mitochondrial dysfunction can induce oligodendrocyte death [36]. Furthermore, oxidative stress has been suggested to be one of the main drivers of demyelination and neurodegeneration in MS [37]. Therefore, blocking oxidative stress during demyelination pathology in MS patients may be a potential therapeutic target.

Phospholipids are an integral part of the composition of myelin and are involved in energy metabolism, regulate biological processes related to learning and memory through several molecular pathways, and are mainly classified as glycerophospholipids and sphingolipids. Sphingolipids are structurally similar to glycerophospholipids, are found in the plasma membrane of most mammalian cells, and are the main components of myelin sheaths [38]. Therefore, alterations in glycerophospholipid metabolism and sphingolipid metabolism pathways may be closely related to the pathological process of CNS demyelinating lesions. We performed metabolic pathway enrichment analysis of differential metabolites by the KEGG database, and the results suggested that these differential metabolites were mainly enriched in the glycerophospholipid and sphingolipid metabolic pathways, so we mainly discussed the nine differential metabolites enriched in these two metabolic pathways. Compared to the controls, among the differential metabolites that were associated with the glycerophospholipid and sphingolipid metabolic pathways, PS (18 : 0/22 : 6(4Z, 7Z, 10Z, 13Z, 16Z, 19Z)), PC (18 : 0/22 : 4(7Z, 10Z, 13Z, 16Z)), and galabiosylceramide (d18 : 1/18 : 0) were increased, while PE (16 : 0/22 : 6(4Z, 7Z, 10Z, 13Z, 16Z, 19Z)), glycerol 3-phosphate, cytidine 5′-diphosphocholine, Cer (d18 : 1/18 : 0), GlcCer (d18 : 1/18 : 0), and SM (d18 : 0/16 : 1(9Z)) were reduced.

Phosphatidylethanolamine (PE) is the second most abundant phospholipid, located on the inner side of the cell membrane and the inner mitochondrial membrane; it participates in immunomodulatory responses [39] and plays a key role in proliferative growth and programmed cell death [38]. In the early stages of apoptosis, PE and phosphatidylserine (PS) are exposed to the outer cell membrane [40] and can induce phagocytosis by macrophages, initiating apoptotic phagocytosis [41, 42]. As a result, the PE (16 : 0/22 : 6(4Z, 7Z, 10Z, 13Z, 16Z, 19Z)) content was reduced in the CPZ group, suggesting that the pathological process of apoptosis and demyelination of oligodendrocytes may be mainly induced by PS. PS is an important component of neuronal cell membranes that can improve nerve cell function, regulate neural electrical signal transmission, and affect membrane fluidity and permeability [43]. The results showed that the content of PS (18 : 0/22 : 6(4Z, 7Z, 10Z, 13Z, 16Z, 19Z)) was significantly higher in the CPZ group. This may be because PS is mainly present on the inner side of mammalian cell membranes, and when exposed on the outer side of the cell membrane, it can act as a trigger for macrophage phagocytosis and initiate cell apoptosis [44]. Cell apoptosis can be triggered by exogenous or endogenous pathways, where oxidative damage and endoplasmic reticulum stress can trigger endogenous apoptosis, and an increased PS content in the outer cell membrane is one of the main features of endogenous apoptosis [45]. Previous studies have found that liposomes bound with PS also enhance phagocytosis of macrophages [46]. Therefore, we speculate that in the CPZ model, the elevated PS content may induce apoptosis of oligodendrocytes, which in turn causes changes in corpus callosum demyelination. Phosphatidylcholine (PC) is the most abundant class of glycerophospholipids in cell membranes and is an integral member in the composition of cell membranes, with the main breakdown products being phosphatidic acid and choline. The results showed a significant increase in the content of PC (18 : 0/22 : 4(7Z, 10Z, 13Z, 16Z)) and a significant decrease in the content of cytidine 5′-diphosphocholine in the CPZ group. It is now widely recognized that oxidative stress is associated with neuronal cell death [47]. The presence of cerebral oxidative stress in neurodegenerative diseases such as Alzheimer's disease and Parkinson's disease has been demonstrated [48, 49], and CPZ interferes with the brain antioxidant system and causes oxidative stress in the corpus callosum. Previous studies have found that PC-specific phospholipase C- (PC-PLC-) catalyzed increases in diacylglycerol (DAG) are associated with the generation of reactive oxygen species (ROS) and that PC-PLC inhibitors can be involved in protecting neurons from oxidative stress [50]. Therefore, the increase in PC content may be the result of compensatory antioxidative stress effects. Cytidine 5′-diphosphocholine is a precursor substance for PC biosynthesis, and the decrease in its content may be due to depletion caused by increased PC synthesis.

Sphingomyelin (SM) plays a key role in the early stages of brain development and is one of the major lipid components of myelin. Ceramide (Cer) is the central metabolite of SM metabolism, and sphingomyelinases (SMases) breakdown SM to Cer and PC. Ceramidase (CDase) further breaks down Cer to sphingosine (Sph), and sphingosine kinase (SphK) cleaves PC to sphingosine phosphate (S1P). SM and its metabolites Cer, Sph, and S1P are important bioactive signaling molecules that can affect immune responses [51]. Villoslada et al. [52] performed a magnetic resonance spectroscopy (MRS) study and found an increase in the phospholipid content and a decrease in the sphingolipid content in the brain tissue of MS patients, and the decrease in CNS myelin-specific lipid content may be related to increased cytoarchitecture due to glial scarring. Our results showed that in the CPZ group, SM in the corpus callosum (d18 : 0/16 : 1(9Z)), Cer (d18:1/18 : 0), and GlcCer (d18:1/18 : 0) levels were reduced in the CPZ group. Glycerol 3-phosphate is a substrate for the synthesis of glycerophospholipids and serves as a potential marker for phospholipid catabolism [53]. In the CPZ group, the corpus callosum glycerol 3-phosphate content was reduced, which may suggest that glycerophospholipid is more anabolic than catabolic in the demyelinated corpus callosum. Galabiosylceramide (Gb2) is synthesized by a-1,4-galactosyltransferase (A4GALT, EC 2.4.1.228), which transmits galactose residues from UDP-galactose to galactosylceramide (GalCer) and can be synthesized from galactosylceramide derived from oligodendrocytes [54]. In the CPZ group, the content of Gab2 was significantly increased. Fabry's disease can be caused by the loss of lysosomal hydrolase *α*-galactosidase, which results in an increase in the Gab2 content [55]. The increase in the Gab2 content in the CPZ group may be due to the abnormal activity of lysosomal hydrolase *α*-galactosidase in oligodendrocytes in the corpus callosum. In addition, the ROC curve results showed that these nine different metabolites had a good degree of discrimination (AUC > 0.75) for the CPZ group, which suggested that the differential metabolites in the glycerophospholipid and sphingolipid metabolic pathways may be potential MS biomarkers.

In conclusion, we performed a metabolomic investigation based on the UPLC-Orbitrap/MS system on the corpus callosum of male mice and identified critical biomarkers and metabolic pathways for CPZ-induced demyelination. The differential metabolism showed high discriminatory capacity for the alteration of demyelination in the corpus callosum. Our results offer new insights into the study of biomarkers and related pathogenesis in the CNS, including MS.

## Data Availability

Data can be provided if necessary.

## Abbreviations

MS: Multiple sclerosis
UPLC-Orbitrap/MS: Ultra-performance liquid chromatography Orbitrap mass spectrometer
LFB: Luxol fast blue
MBP: Myelin basic protein
GFAP: Glial fibrillary acidic protein
IPA: Ingenuity pathway analysis
CPZ: Cuprizone
CON: Control
SD: Standard deviation
ANOVA: One-way analysis of variance
ROC: Receiver operating characteristic
VIP: Variable influence on projection
TIC: Total ion flow chromatogram
PCA: Principal component analysis
PLS-DA: Partial least squares discriminant analysis
PE: Phosphatidylethanolamine
PS: Phosphatidylserine
PC: Phosphatidylcholine
PC-PLC: PC-specific phospholipase C
DAG: Diacylglycerol
ROS: Reactive oxygen species
SM: Sphingomyelin
Cer: Ceramide
SMase: Sphingomyelinases
CDase: Ceramidase
Sph: Sphingosine
SphK: Sphingosine kinase
S1P: Sphingosine phosphate
MRS: Magnetic resonance spectroscopy
Gb2: Galabiosylceramide
GalCer: Galactosylceramide.
